# Enhanced hippocampal neurogenesis mediated by PGC-1α-activated OXPHOS after neonatal low-dose Propofol exposure

**DOI:** 10.3389/fnagi.2022.925728

**Published:** 2022-07-27

**Authors:** Keyu Chen, Dihan Lu, Xiaoyu Yang, Rui Zhou, Liangtian Lan, Yan Wu, Chen Wang, Xuanxian Xu, Mei Hua Jiang, Ming Wei, Xia Feng

**Affiliations:** ^1^Department of Anesthesiology, First Affiliated Hospital, Sun Yat-sen University, Guangzhou, China; ^2^Key Laboratory for Stem Cells and Tissue Engineering, Center for Stem Cell Biology and Tissue Engineering, Ministry of Education, Zhongshan School of Medicine, Sun Yat-sen University, Guangzhou, China; ^3^Department of Hepatobiliary Surgery, Sun Yat-Sen Memorial Hospital, Sun Yat-sen University, Guangzhou, China; ^4^Department of Anatomy, Zhongshan School of Medicine, Sun Yat-sen University, Guangzhou, China; ^5^Guangdong Key Laboratory of Reproductive Medicine, Guangzhou, China; ^6^Program of Stem Cells and Regenerative Medicine, Affiliated Guangzhou Women and Children's Hospital, Zhongshan School of Medicine, Sun Yat-sen University, Guangzhou, China

**Keywords:** Propofol, hippocampal neurogenesis, neural stem cell, OXPHOS, PGC-1α

## Abstract

**Background:**

Developing brain is highly plastic and can be easily affected. Growing pediatric usage of anesthetics during painless procedures has raised concerns about the effect of low-dose anesthetics on neurodevelopment. It is urgent to ascertain the neuronal effect of low-dose Propofol, a widely used anesthetic in pediatrics, on developing brains.

**Methods:**

The behavioral tests after neonatal exposure to low-dose/high-dose Propofol in mice were conducted to clarify the cognitive effect. The nascent cells undergoing proliferation and differentiation stage in the hippocampus and cultured neural stem cells (NSCs) were further identified. In addition, single-nuclei RNA sequencing (snRNA-seq), NSCs bulk RNA-seq, and metabolism trials were performed for pathway investigation. Furthermore, small interfering RNA and stereotactic adenovirus injection were, respectively, used in NSCs and hippocampal to confirm the underlying mechanism.

**Results:**

Behavioral tests in mice showed enhanced spatial cognitive ability after being exposed to low-dose Propofol. Activated neurogenesis was observed both in hippocampal and cultured NSCs. Moreover, transcriptome analysis of snRNA-seq, bulk RNA-seq, and metabolism trials revealed a significantly enhanced oxidative phosphorylation (OXPHOS) level in NSCs. Furthermore, PGC-1α, a master regulator in mitochondria metabolism, was found upregulated after Propofol exposure both *in vivo* and *in vitro*. Importantly, downregulation of PGC-1α remarkably prevented the effects of low-dose Propofol in activating OXPHOS and neurogenesis.

**Conclusions:**

Taken together, this study demonstrates a novel alteration of mitochondrial function in hippocampal neurogenesis after low-dose Propofol exposure, suggesting the safety, even potentially beneficial effect, of low-dose Propofol in pediatric use.

## Introduction

The early stage of life is believed to be the vulnerable period of brain development. Early life events can exert a powerful influence on both the pattern of brain architecture and behavioral development (Jevtovic-Todorovic and Brambrick, 2018). With the growing number of infants and children exposed to general anesthesia or sedation for surgery or examinations, the effect of anesthetics on brain development remains controversial. Anesthesia-induced neurotoxicity in the developing brain has been revealed in numerous studies (Vutskits and Xie, 2016; Zhou et al., 2021; Ing et al., 2022), while the neuroprotective property of general anesthetics, especially low-dose anesthetics, is increasingly recognized (Li et al., 2016; Wu et al., 2019; Zhao et al., 2022). As previously described, the application of low-dose ketamine could enhance neurogenesis in mice in the depression model (Deyama and Duman, 2020) and showed significant antidepression effects in human studies (Colla et al., 2021). Besides, a low dose of sevoflurane has been reported to stimulate neurogenesis and promote learning and memory ability in neonatal rats (Chen et al., 2015). However, the effects of low-dose Propofol, which has been commonly used in sedation for non-invasive procedures, on neurodevelopment and cognitive function in the developing brain remain uncovered.

Hippocampal neurogenesis is the crucial process of neurodevelopment, which is well known involved in determining emotion and cognitive function (Anacker and Hen, 2017). This includes the process of neural stem cells (NSCs) proliferation, differentiation into neuronal progenitor cells (NPCs), and giving rise to granule neurons (GCs) in the dentate gyrus (DG) (Toda et al., 2019). Highly activated neurogenesis in the young mammalian brain could be affected by specific factors, such as epigenetic changes, metabolic shift in NSCs, extracellular matrix signaling, neuropeptides, and other neurogenesis-related transcription factors (Vieira et al., 2018; Niklison-Chirou et al., 2020). Mitochondrial metabolism, including oxidative phosphorylation (OXPHOS), has been reported to mediate neurogenesis and NSCs fate decision (Khacho et al., 2019). It had been reported that efforts to enhance mitochondrial function can improve impaired neurogenesis and behavioral deficits in young mice after neonatal hypoxia exposure (Sun et al., 2020).

In this study, we aimed to determine the effects of neonatal exposure to low-dose Propofol on cognitive function as well as altered neurogenesis state. Furthermore, single-nuclei RNA-seq (snRNA-seq) and NSCs bulk RNA-seq analysis were used for mechanism investigation and further experiments were conducted to verify the pathway. This study might suggest the effect and mechanisms of low-dose Propofol on neurodevelopment, and its potential benefits in clinical pediatrics sedation.

## Materials and methods

### Animals

C57BL/6N mice were purchased from the Beijing Vital River Laboratory Animal Technology Co., Ltd. (Beijing, China). Homozygous Nestin-GFP transgenic mice that express enhanced GFP under the control of the Nestin promoter (Nes-GFP), which were on the C57BL/6J genetic background, were kindly provided by Yamaguchi et al. (2000) as previously reported. All the mice were housed in a room at 23°C under a 12-h (h) light/dark cycle and were given food and water *ad libitum*. All animal protocols were reviewed and approved by the Sun Yat-Sen University Institutional Animal Care and Use Committee. Newborn mice from the maternal mouse, both male and female mice, would be included in the experiment and divided into groups randomly with an online randomization tool: http://www.randomizer.org. Mice injured severely would be excluded.

### Drug administration and EdU labeling *in vivo*

Wild-type C57BL/6N mice or Nestin-GFP mice at postnatal day (PND) 7 were randomly allocated to different groups and administrated 4 mg.kg^−1^ (L-Propofol group) and 50 mg.kg^−1^ (H-Propofol group) Propofol (Diprivan; AstraZeneca; Cambridge, England) by i.p. injection. EdU (5-ethynyl-2'-deoxyuridine; A10044; Invitrogen; California, USA) was first given i.p. 30 min after drug injection at a dose of 50 mg kg^−1^ in 0.9% saline and the same dosage was administrated per 12 h for six times within 3 days.

### Tissue processing

Tissues were obtained from mice sacrificed on the 3-day post-injection (dpi), 7 dpi, and 28 dpi of Propofol injection for immunofluorescent staining. For positive cell counting, at least three hippocampal sections from three different individuals were calculated. Sections were paired with the same location of the hippocampus among groups. For 10× Genomics snRNA-seq, hippocampus were dissociated freshly from PND10 mice brain 3 dpi of propofol.

### Primary NSCs separation, culture, and induction

NSCs were obtained from cortical tissues collected from C57BL/6N mouse fetuses on embryonic day 14.5. The dissociated cells were seeded in the NSCs medium, DMEM/F12 (SH30023.01; HyClone; Logan, Utah, USA) supplemented with epidermal growth factor (EGF) and basic fibroblast growth factor (bFGF) (both at 20 ng ml^−1^; 100-47, 100-18B; Peprotech; Cranbury, NJ, USA), B27 (2%; 17504-044; Gibco), N2 (1%; 17502-048; Gibco; Grand Island, NewYork, USA), and 100 IU/ml penicillin/streptomycin (SV30010; HyClone) to form primary neurospheres and grown in 5% CO2 in the air, at 37°C. NSCs differentiation was induced by plating cells onto 24-well plates in DMEM/F12 medium supplemented with 2% FBS (10099; Invitrogen), 2% B27, and 100 IU.ml^−1^ penicillin/streptomycin.

### Treatment of the primary NSCs

NSCs in passages 4 to 6 were seeded into cell culture plates with PLL (poly-L-lysine; P4707; Sigma) and incubated overnight. About 10 μM Propofol (1.78 μg.ml^−1^, deemed as low-dose Propofol) was added to the cells for 24 h as previously described (Qiao et al., 2017). For proliferation tests, the cell numbers were counted once daily for consecutive 6 days in 24-well plates. For differentiation tests, the cells were maintained in a differentiation medium for 10 days before being analyzed by Tuj-1 (neuron) and GFAP (astrocyte) staining. The antibodies used are shown in the Supplementary material. For NSCs bulk RNA-seq, cells were harvested 24 h after 10 μM Propofol treatment.

### 10× genomics snRNA-seq

#### Nucleus dissociation

Hippocampus nucleus was isolated at 4°C. The tissue was homogenized using a douncer with Nuclei EZ Lysis Buffer (Nuc101, Sigma) in the tube. After incubated with Nuclei EZ Lysis Buffer on ice, the homogenate was filtered with a 70-μm strainer mesh. The sample was centrifuged at 500 g for 5 min and was resuspended pellet with another EZ Lysis buffer. After incubated for 5 min on ice, the sample was centrifuged at 500 g for 5 min. Nuclei Wash and Resuspension Buffer were added to the pellet and incubate for 5 min. After incubation, another Nuclei Wash and Resuspension Buffer are added and the nucleus was resuspended. Centrifuged the nucleus at 500 g for 5 min and resuspended with Nuclei Wash and Resuspension Buffer supplemented with DAPI. Collected all nuclei by filtering with a 35-μm strainer. The number of nuclei was assessed under a microscope and counted with a cell counter. Sorted nucleus into 10× RT Buffer prepared without the RT Enzyme Mix and proceeded with the 10× Genomics Single Cell Protocol.

#### cDNA library construction and sequencing

Gel Beads-In-Emulsions (GEMs) were performed by loading onto the Chromium Controller (10× Genomics) following the manufacturer's instruction (Chromium Single Cell 3' Library & Gel Bead Kit v2). The amplified cDNA was optimized by enzymatic fragmentation and size selection before library construction. The single-cell libraries were sequenced on a HiSeq4000 system (Illumina).

#### snRNA-seq data analysis

Bioinformatic analysis of snRNA-seq was conducted by LC-bio (Hangzhou, China). The data were post-processed and quality controlled using the 10× Cell Ranger package (v1.2.0; 10× Genomics). Reads were demultiplexed, aligned, and quantified by the 10× Cell Ranger (version 6.1.1). Aggregate options were analyzed with the R package Seurat (version 3.0) with default parameters. Cells were considered valid only when genes were more than 200 and <2,500 as well as containing <5% mitochondrial genes. A correlation analysis was performed by employing the RunPCA function of the Seurat package. Clustering analysis was carried out with standard Seurat package procedures with a resolution of 1.2. Data visualization was performed using Uniform Manifold Approximation and Projection (UMAP) of the principal components in Seurat. Pseudotime analysis was used to illustrate the transition of neurogenic lineage cells during neurogenesis by importing the data to Monocle2 to perform cell trajectory analysis. To investigate the potential effect of low-dose Propofol on cell clusters, we calculated gene expression. Differentially expressed genes (DEGs) were identified by the Seurat bimod test (Seurat2.3.4) according to the literature (Ximerakis et al., 2019) [*p*-value < 0.05 and log2-fold change (log2FC) ≥ 0.138].

### Bulk RNA-seq

Total RNA from the NSCs was isolated using the TRIzol reagent. The purity and quantity of the total RNA were evaluated using a NanoDrop1000 spectrophotometer (Thermo Fisher Scientific Inc.). The absorbance ratio of OD260/280 was between 1.8 and 2.0 for all samples, and RNA integrity was assessed by electrophoresis analysis on a standard denaturing agarose gel. The library for transcriptome sequencing was conducted by NovelBio Co., Ltd (Shanghai, China). DEGs were screened by the principles: log2FC > 0.585 or < −0.585, FDR < 0.05. GO analysis was applied to analyze the main function of the differential expression genes according to the Gene Ontology from NCBI. Generally, Fisher's exact test was applied in this study to identify the significant GO pathways and the false discovery rate (FDR) was used to correct the *p*-values. Thus, based on the FDR value, the top 10 pathways were included for evaluation. GO pathways presentation was based on the ratio of the upregulated DEGs in total different genes.

### siRNA transfection of NSCs

PGC-1α siRNA and control siRNA were obtained from the RiboBio company (Guangzhou, China). The PGC-1α-specific siRNAs were 5′ -CUGCGAACAUAUUUGAGAA dTdT-3′ (sense) and 5′ -UUCUCAAAUAUGUUCGCAG dTdT-3′ (antisense). About 50 μM siRNA diluted in Opti-MEM medium (Invitrogen, 13778075, Carlsbad, CA) and Lipofectamine RNAimax transfection reagent (Invitrogen, 11058021, Carlsbad, CA) were used according to the manufacturer's protocol for transfection. After seeding for 24 h, the cells were transfected with siRNA and cultured in an NSC medium for 8 h. Cells were cultured in an NSCs differentiation medium for 1 and 4 days for qPCR and staining, respectively.

### Stereotactic virus injection

The PGC-1α shRNA and control shRNA ligated into adenovirus vector expressing EGFP were obtained from WZ Biosciences Inc. (Shandong, China). PGC-1α-specific shRNA were CTGCGAACATATTTGAGAATTCAAGAGATTCTCAAATATGTTCGCAGTTTTTT. We made a tailored stage for stereotactic injection according to the literature (Mathon et al., 2015). After anesthetized by a freeze, PND3 mice were placed into the tailored stage on a stereotactic frame (KOPF, KD Scientific) for injection. Scissors were used to make a 5-mm incision to make the skull visible. After determining the lambda, find the coordinate position (anterior-posterior, +1.1 mm, mediolateral, ±0.9 mm, dorsoventral, −2.0 mm referring to lambda) and directly pinch with the Hamilton syringe (Hamilton Medical, Reno, NV, USA). The virus was injected into the hippocampus with 0.12 μl of viral titer (1.2 × 10^9^ vg. ml^−1^) at an injection speed of 0.12 μl min^−1^. The injections were conducted in PND3 mice and immunofluorescence staining was performed on PND10 by calculating infected cells and PGC-1α intensity in DG to evaluate the efficiency.

### Other methods

Procedures related to the behavioral test, immunofluorescent staining, apoptosis analysis, western blot analysis, qRT-PCR analysis, HPLC analysis, and seahorse metabolism analysis are provided in the Supplementary material.

### Statistical analysis

All results were obtained from at least three independent experiments and expressed as mean ± SD. Student's *t*-test was used to compare the means between two groups, while one-way ANOVA was performed for comparisons of three or more groups, followed by an LSD test for multiple comparisons. One-way ANOVA repeated measurement analysis was used to compare repeated assessments. The *p* < 0.05 was considered significant. Data of snRNA-seq was analyzed on the website: https://www.omicstudio.cn. Statistical analysis and graphs generation were performed using SPSS Statistics 26 (Armonk, New York, U.S.), GraphPad Prism software 9 (San Diego, CA, USA), and website: https://app.biorender.com.

## Results

### Low-dose Propofol enhanced spatial cognitive ability in neonatal mice

**Figure 1:**
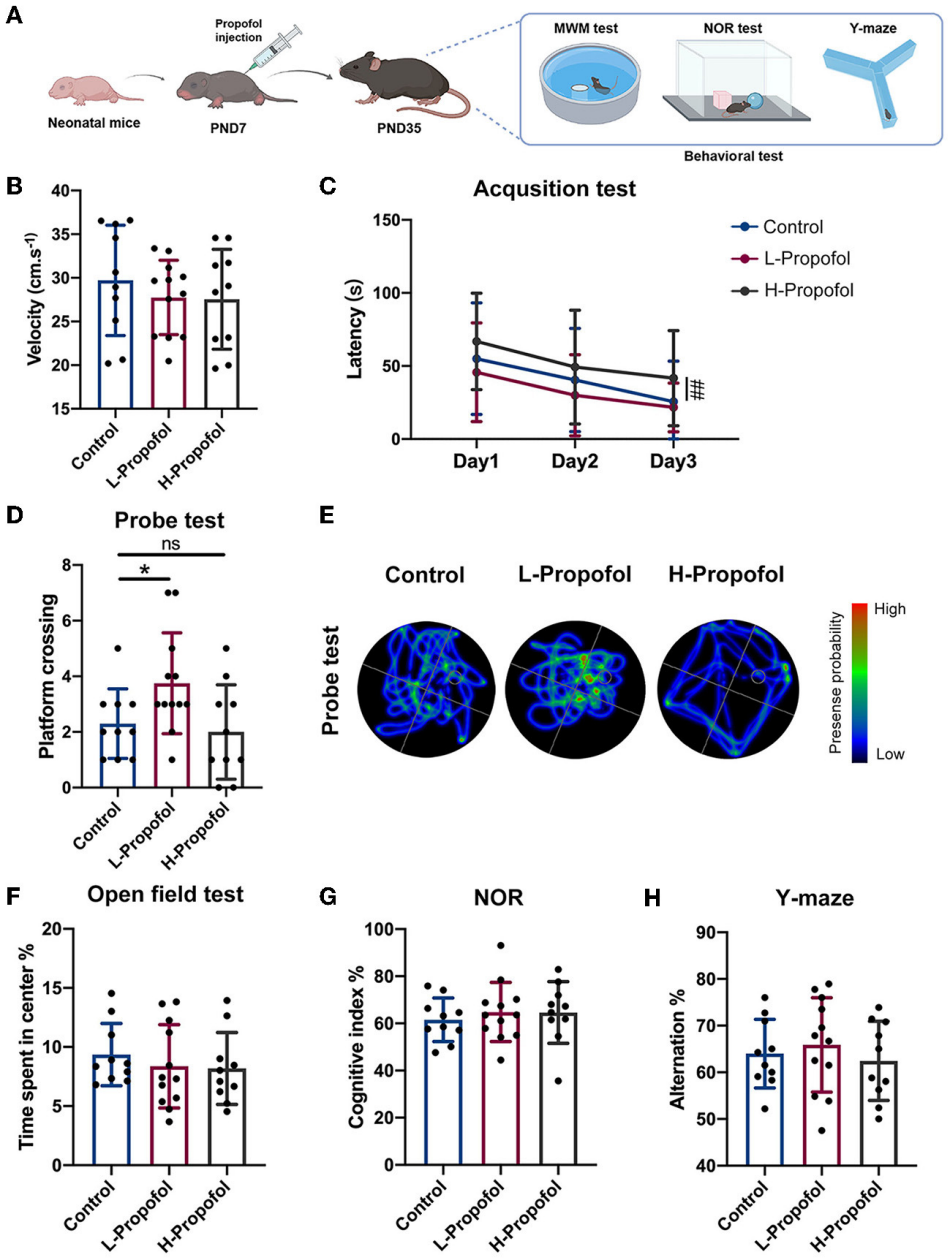
Early low-dose Propofol administration promoted spatial cognitive ability in mice. **(A)** Workflow of Propofol treatment and behavior tests in C57BL/6N. **(B)** Velocity of mice among groups showing similarity on day 1 of acquisition test. **(C)** Acquisition test showing a trend that L-Propofol mice spent less time on the hidden platform compared with the Control group, while H-Propofol mice exhibited increased escape latency (H-Propofol vs. Control: *p* = 0.009) relative to the other two groups during day 1–3. **(D)** Platform crossing in probe test showing that L-Propofol mice significantly increased the number of platform crossing (L-Propofol vs. Control: *p* = 0.046). **(E)** Typical swimming path in the probe test. **(F)** Time spend exploring the center in OFT. **(G)** The cognitive index in NOR. **(H)** The percentage of spontaneous alternation in Y-maze. Three groups performed similarly in OFT, NOR, and Y-maze. *N* = 10 (Control), 12 (L-Propofol), 10 (H-Propofol). Error bars means ± SD. * (L-Propofol vs Control), *p* < 0.05; *##* (H-Propofol vs Control), *p* < 0.01, ns, no significant. One-way repeated measures ANOVA, one-way ANOVA, and LSD test for multiple comparisons were used for analysis.

To uncover the effect of different doses of Propofol on cognitive function, 4 mg.kg^−1^ (L-Propofol, sub-anesthesia dose reported) (Chen et al., 2021) and 50 mg.kg^−1^ (H-Propofol, anesthesia dose used previously) (Jiang et al., 2018) of Propofol were delivered by intraperitoneal (i.p.) injection on PND 7 C57BL/6N mice, and behavioral tests were conducted according to the workflow (Figure 1A).

Morris water maze (MWM) was performed to clarify the effect on spatial learning and memory ability since PND35. There was no motor deficit for the similar velocity of swimming among groups during the training session (Figure 1B). Curiously, improved spatial cognitive function was observed in the L-Propofol group with a shortening trend of escape latency during the acquisition trial as well as significantly more platform crossing in the probe test (1.6-fold) (Figures 1C–E). Besides, plasma concentrations of mice in the L-Propofol group were measured and verified as clinically sedative concentrations (Supplementary Figure 1, 1.36 μg.ml^−1^) (Kim, 2007). Nevertheless, impaired learning ability was confirmed in the H-Propofol group for significantly longer escape latency (Figures 1C–E). In addition, we further assessed the effects of Propofol on anxiety emotion with an open field test (OFT), recognition memory *via* novel object recognition test (NOR), and working memory by Y-maze. No significant difference was observed among the three groups in these tests (Figures 1F–H). Taken together, these results implicated that low-dose Propofol improved spatial cognitive function specifically, with no obvious change in object recognition memory, working memory, or emotion-related performance. Thus, the L-Propofol group was adopted for the following experiments.

### Low-dose Propofol-treatment activated hippocampal neurogenesis process both *in vivo* and *in vitro*

**Figure 2:**
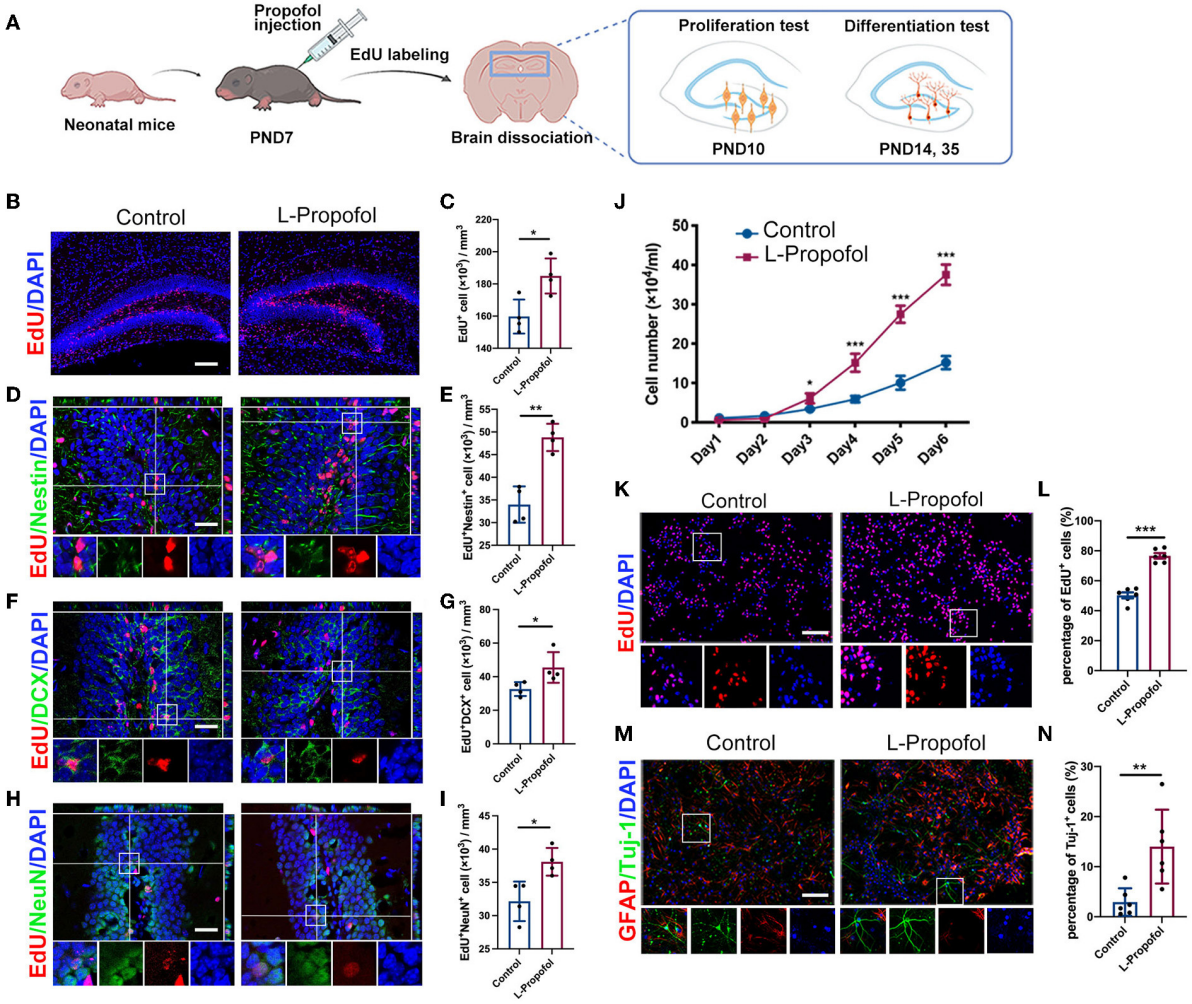
Long-term tracing showed boosted hippocampal neurogenesis after low-dose Propofol treatment. **(A)** Timeline for treatment for hippocampal neurogenesis. **(B,C)** Images of maximum intensity projection **(B)** and quantification **(C)** showing the increased EdU^+^(red) cells (*p* = 0.016) in DG of mice at PND 10 in the L-Propofol group. *N* = 4. Scale bars, 200 μm. **(D,E)** Images with z-plane projections **(D)** and quantification **(E)** showing the increased Nestin^+^ (green)/EdU^+^ (red) newborn NSCs (*p* = 0.001) in DG of mice at PND 10 in the L-Propofol group. *N* = 4. **(F,G)** Images with z-plane projections **(F)** and quantification **(G)** showing an increased DCX^+^ (green)/EdU^+^ (red) newborn neurons (*p* = 0.043) in the DG of mice at PND 14 in the L-Propofol group. *N* = 4. **(G,I)** Images with z-plane projections **(H)** and quantification **(I)** showing an increased NeuN^+^ (green)/EdU^+^ (red) mature neurons (*p* = 0.017) in the DG of mice at PND 28 in the L-Propofol group. *N* = 4. For **(D–I)**, Scale bars, 50 μm. Bottom panels: higher-magnification views of selected regions. **(J)** Growth curves of NSCs as assessed by direct counting for consecutive 6 days after treatment (*p* < 0.001). *N* = 3. **(K,L)** Fluorescence images **(K)** and quantification analysis **(L)** showing significantly increased EdU^+^ cells in NSCs (*p* < 0.001) at 4 days after treatment. Scale bar: 50 μm. *N* = 6. **(M,N)** Fluorescence staining **(M)** and quantification **(N)** showing an increased proportion of Tuj-1^+^ newborn neurons (*p* = 0.006) at 10 days after differentiated induction in the L-Propofol group. *N* = 6. Scale bar: 20 μm. Error bars, mean ± SD, Bottom panels: higher-magnification views of selected regions. *p < 0.05, **p < 0.01, ***p < 0.001. Repeated measurement and Student's *t*-test were used for analysis.

Since hippocampal neurogenesis contributes to specific spatial cognitive function (Goncalves et al., 2016), lineage tracing with EdU labeling was used to assess the proliferation and differentiation of NSCs (Figure 2A). Quantitative analysis showed significantly expanded EdU^+^ cells (1.2-fold) in DG 3 dpi of Propofol (Figures 2B,C), suggesting increased hippocampal newborn cells in neonatal mice after exposure to low-dose Propofol. Further analysis revealed increased EdU^+^/Nestin^+^ cells (1.4-fold) at 3 dpi indicating NSCs expansion in the L-Propofol group (Figures 2D,E). To clarify the fate of the expanded cells, we next assessed EdU^+^/DCX^+^ cells representing newborn neurons at 7 dpi and EdU^+^/NeuN^+^ cells representing mature neurons at 28 dpi. Consequently, significantly elevated neuronal fate (1.4-fold and 1.2-fold) was found in the L-Propofol group (Figures 2F–I). All of these data demonstrated the activated hippocampal neurogenesis after low-dose Propofol administration, which might be involved in enhanced spatial cognitive capability.

To elucidate the explicit change in NSCs after low-dose Propofol treatment, primary-cultured NSCs were obtained and an overall activation was observed with 1 to 100 μM Propofol (Supplementary Figure S2). We adopted the clinical-related concentration of 10 μM (Schraag et al., 2019) and deemed it as low-dose Propofol for our following study. Similar to the effect *in vivo*, low-dose Propofol treatment markedly increased the number of viable cells (Figure 2J) and EdU-incorporating cells (1.5-fold) (Figures 2K,L) and without resulting in apoptosis level alternation (Supplementary Figure 3), indicating the promoted NSCs proliferation after low-dose Propofol treatment. Besides, treatment with low-dose Propofol significantly increased the number of Tuj-1^+^ cells (4.7-fold) in the NSCs differentiation assay (Figures 2M,N). In summary, the results suggested that low-dose Propofol might simultaneously enhance both proliferation and neuronal differentiation of NSCs *in vitro*.

In addition, we further assessed adult neurogenesis in 18-month-old mice in Control and L-Propofol groups to verify whether L-Propofol-activated hippocampal neurogenesis results in diminished NSCs pool with aging (Ceccarelli et al., 2020). The number of NSCs storage was examined by immunohistological staining with Sox2, a transcription factor important for controlling NSCs maintenance (Favaro et al., 2009). Similar Sox2 expression was observed in both Control and L-Propofol groups, implying no obvious persistent hazard during aging after neonatal low-dose Propofol exposure (Supplementary Figure 4).

### snRNA-seq showed enhanced hippocampal neurogenesis in 3-day post-treatment neonatal mice

**Figure 3:**
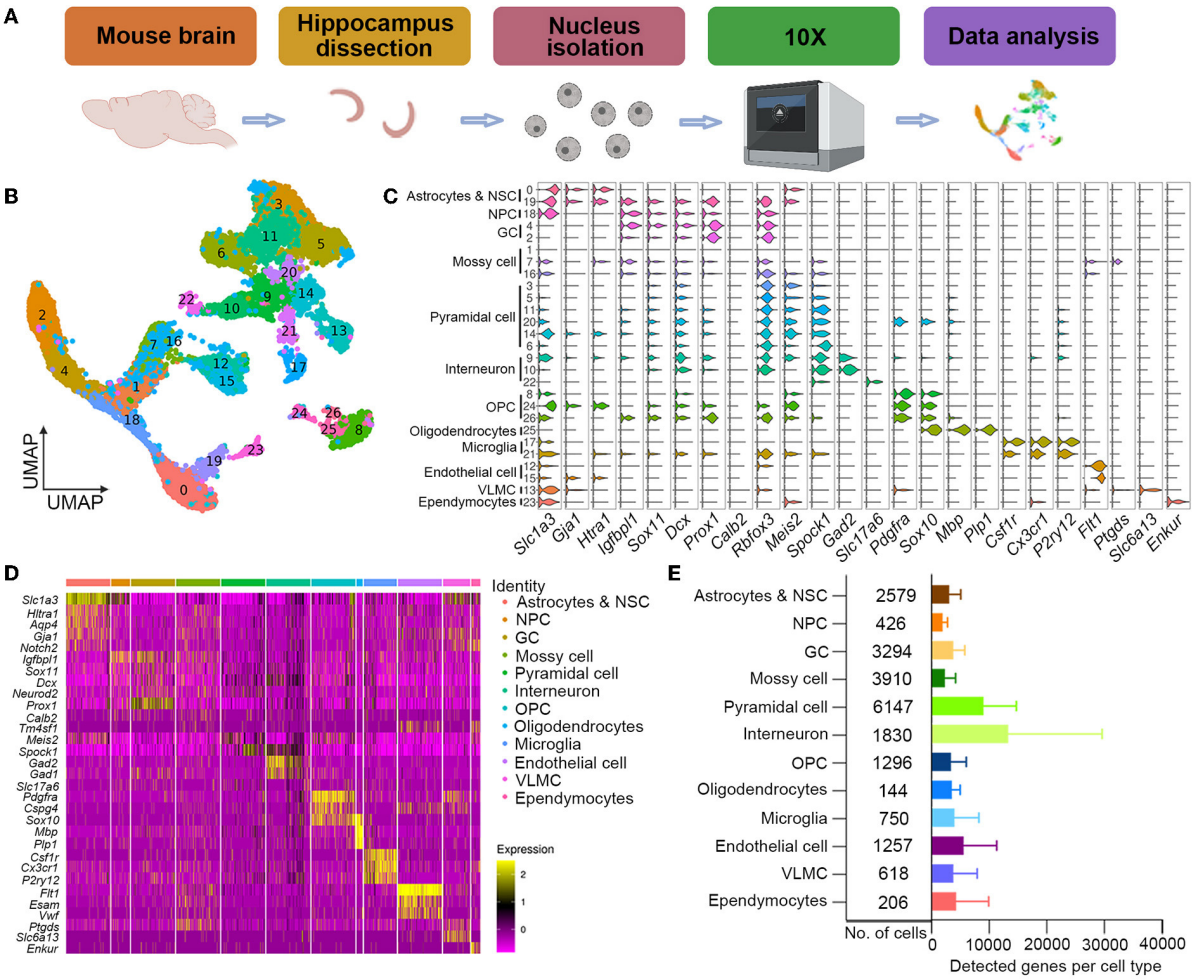
Single-nuclei cell transcriptome atlas of hippocampal neurogenesis after low-dose Propofol exposure. **(A)** Schematics of single-nuclei RNA sequence experiment and data analysis. **(B)** UMAP visualization integrating 22,457 high-quality single cells from hippocampus samples at PND 10 after exposure, colored by cluster annotation. **(C)** Violin plot showing the distribution of expression levels of representative cell-type-enriched marker genes across 12 cell types. The x-axis indicates the normalized expression levels of the corresponding marker genes. **(D)** Heatmap of all single cells ordered by UMAP clusters. Columns, individual cells. Rows, genes. Boxed areas, gene expression modules. **(E)** Bar plot showing the total number of detected cells and the average number of detected genes per cell type. Error bars, mean ± SD.

To characterize the variation of neurogenesis-related cell types affected by low-dose Propofol precisely and illustrate the molecular signatures, we carried out snRNA-seq, which is particularly suited to investigate neuronal development (Armand et al., 2021), in the hippocampus at 3 dpi in neonatal mice. A total of 22,457 (11,347 in Control and 11,110 in L-Propofol) nuclei were isolated and a 10× Genomics platform was used for single-nuclei transcriptomes (Figure 3A). UMAP was used to separate the dataset into 26 clusters (Figure 3B) and subsequently classified the major clusters into 12 cell types (Figures 3C–E): astrocytes and NSCs (expressing Hopx, Notch2, and Aqp4), NPCs (expressing Igfbpl1 and Sox11), GCs (expressing Prox1), mossy cells (expressing Tm4sf1 and Calb2), pyramidal cells (expressing Meis2 and Spock1), interneurons (expressing Gad1, Gad2, and Slc17a6), oligodendrocyte precursor cells (OPCs, expressing Pdgfra), oligodendrocytes (expressing Mbp), microglia (expressing Cx3cr1), endothelial cells (expressing Flt1), vascular and leptomeningeal cells (VLMCs, expressing Slc6a13), and ependymocytes (expressing Enkur), consistent with previous studies (Cembrowski et al., 2016; Artegiani et al., 2017; Hochgerner et al., 2018; Rosenberg et al., 2018; Zywitza et al., 2018; Mizrak et al., 2019).

**Figure 4:**
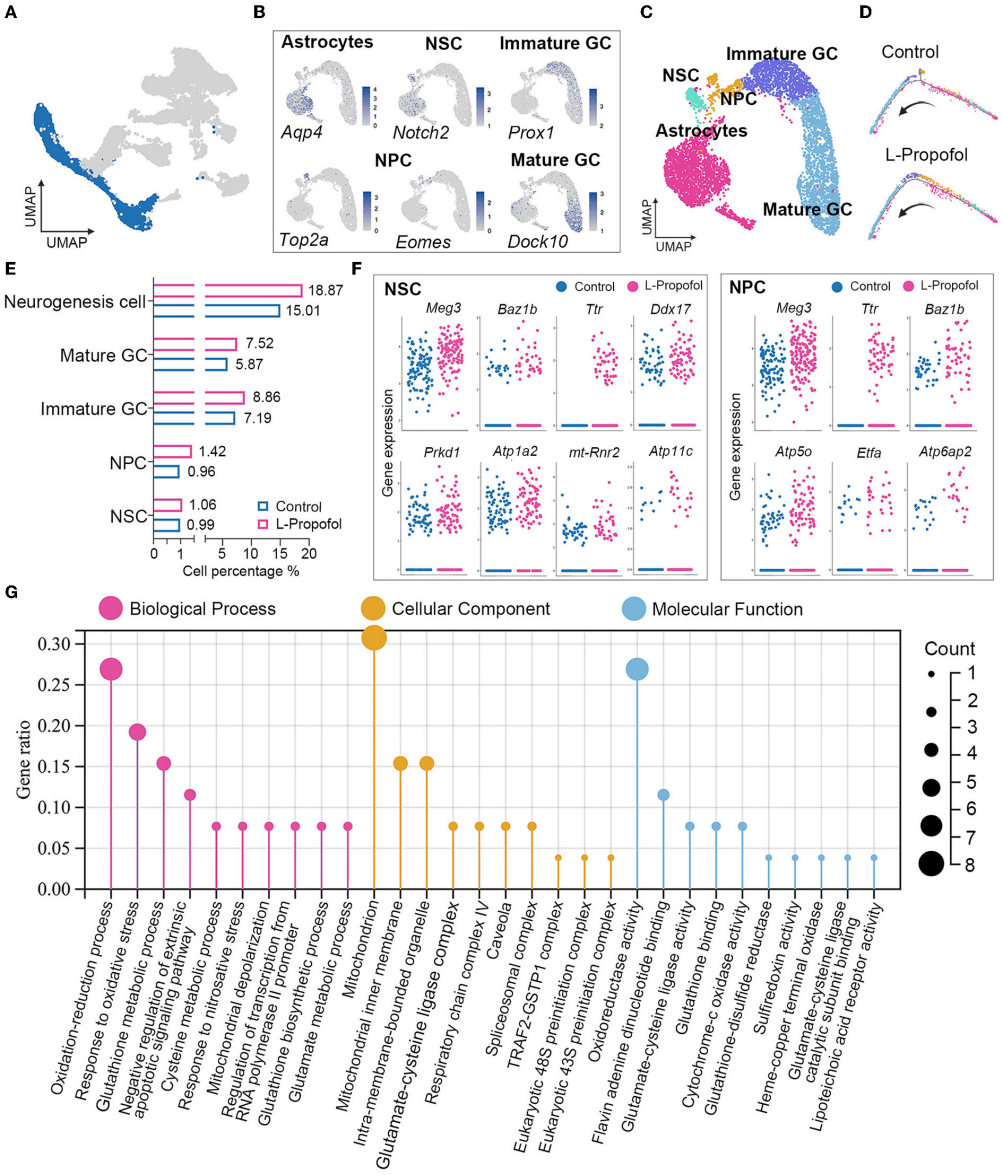
Enhancement of mitochondria metabolism in NSCs post-treatment revealed by transcriptome analysis. **(A)** Target clusters of neurogenesis-related cell types for cell regrouping. **(B)** Expression of representative markers for each cell type. Cells were colored according to their expression levels, ranging from “not detected” (gray) to “highest detected” (blue). **(C)** Distribution for neurogenesis-related cell types in UMAP analysis in which clusters were separated by colors. **(D)** Pseudotime analysis of neurogenesis in Control group and L-Propofol group, respectively. **(E)** Quantification of the percentage of NSCs, NPCs, immature GCs, mature GCs, and overall neurogenesis cells among all cells showing overall activated neurogenesis in the L-Propofol group. **(F)** Level of gene expression showing the enhancement of neurogenesis and mitochondrial metabolism-related genes in NSCs (Meg3: *p* < 0.001, Ttr: *p* < 0.001, Baz1b: *p* = 0.01, Ddx17: *p* = 0.03, Atp1a2: *p* < 0.001, Prkd: *p* = 0.04, mt-Rnr2: *p* < 0.001, Atp11c: *p* = 0.04) and NPCs (Meg3: *p* < 0.001, Ttr: *p* < 0.001, Baz1b: *p* < 0.001, Atp5o: *p* = 0.01, Etfa: *p* = 0.03, Atp6ap2: *p* < 0.001) between Control and L-Propofol. **(G)** GO analysis showing the mitochondrial metabolism pathway enrichment in NSCs in the L-Propofol group compared with the Control group.

To assess the overall effect of low-dose Propofol on cell fate specification, we regrouped neurogenesis-related cells for the difficulty in differing astrocytes and NSCs in the primary classification as the similar phenotype with sharing markers (Artegiani et al., 2017). Thus, neurogenesis-related cell types including astrocytes and NSCs, NPCs, and GCs were re-separated into five clusters (Figures 4A–C): astrocytes (expressing Aqp4), NSCs (expressing Notch2), NPCs (expressing Top2a and Eomes) (Hochgerner et al., 2018), immature GCs (expressing Prox1), and mature GCs (expressing Dock10). Pseudotime analysis suggested that the development of neurogenesis-related cells between groups was similar (Figure 4D). In addition, the ratios of cells in each cluster over total cells in the hippocampus were calculated, and the result showed an increasing trend in the percentages of neurogenesis-related cells in the L-Propofol group, including an 8% increase in NSCs, 48% increase in NPCs, 23% increase in immature GCs, and 28% increase in mature GCs (Figure 4E).

### Transcriptome analysis revealed mitochondrial metabolism enhancement in hippocampal NSCs after low-dose Propofol treatment

To further study the dynamic change of hippocampal NSCs after low-dose Propofol administration, significantly differential genes and transcriptome analysis were performed in early neurogenesis-related cell types including NSCs and NPCs according to the clusters revealed by snRNA-seq. A total of 1,975 total genes were detected and 865 were identified as significantly affected in the L-Propofol group at least in one cell type. Further analysis of significantly upregulated genes revealed that low-dose Propofol exposed NSCs and NPCs presented higher expression (1.1 to 2.4-fold) of neurogenesis promotion-related genes such as Meg3, Ttr, Baz1b, Ddx17, and so on (Kapoor et al., 2015; Lalli et al., 2016; Gao et al., 2019; Suthapot et al., 2022), and mitochondrial metabolism-related genes such as Atp1a2, mt-Rnr2, Atp11c, Atp5o, Etfa, Atp6ap2, and so on (Figure 4F). Besides, GO analysis of NSCs bulk RNA-seq obtained 24 h after low-dose Propofol administration revealed the enrichment in mitochondrial metabolism (Figure 4G). The results suggested that low-dose Propofol might promote mitochondrial metabolism in hippocampal NSCs.

**Figure 5:**
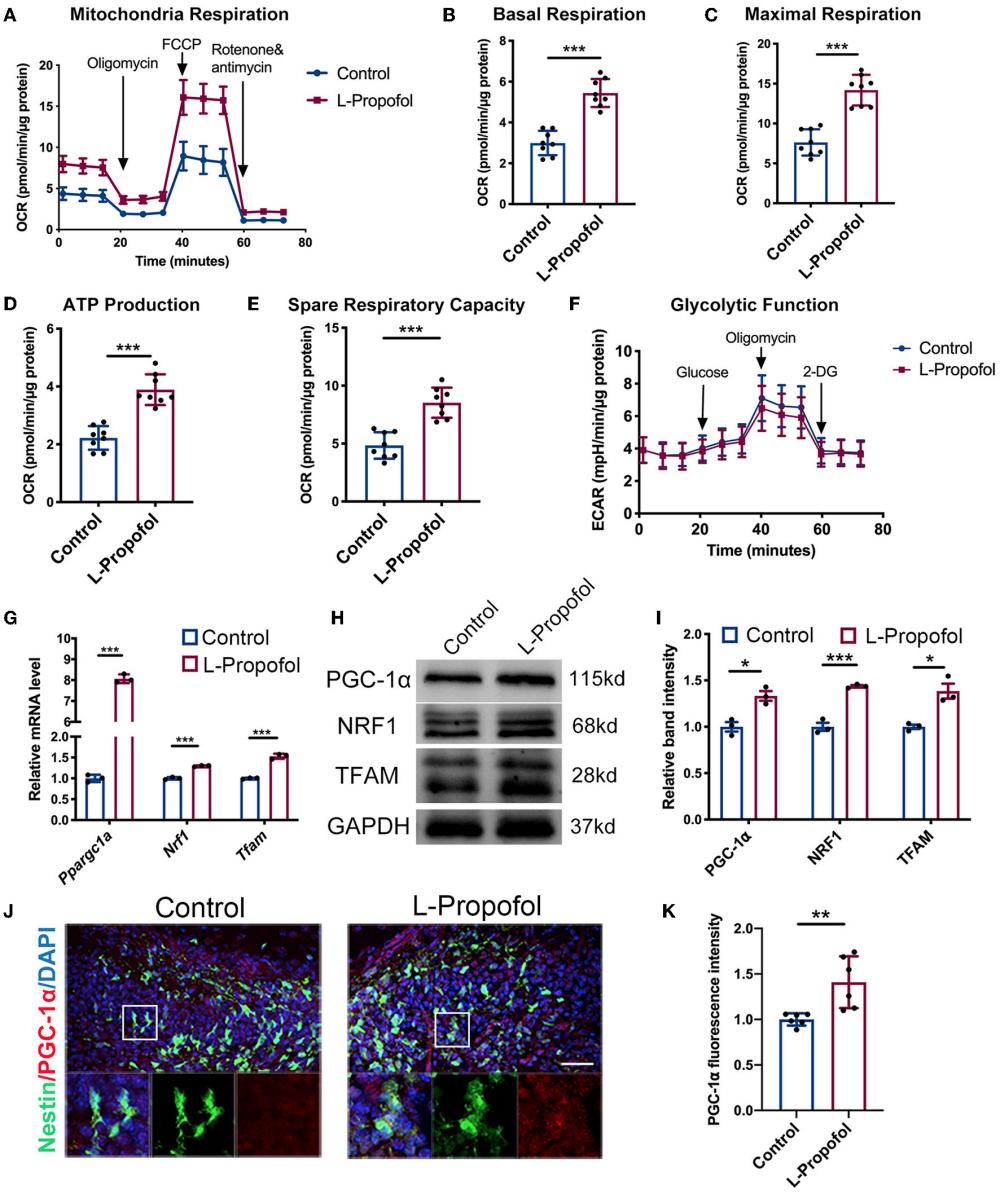
Metabolic shift and activated PGC-1α/NRF1/TFAM pathway in NSCs were confirmed after Propofol treatment. **(A–E)** Low-dose Propofol enhanced OXPHOS level in primary NSCs measured by the **(A)** oxygen consumption rate (OCR) normalized by protein content (μg) including OXPHOS parameters **(B)** basal respiration (*p* < 0.001), **(C)** maximal respiration (*p* < 0.001), **(D**) ATP-linked respiration (*p* < 0.001), and **(E)** spare respiratory capacity (*p* < 0.001). *N* = 8. **(F)** Glycolytic function showed a similar result in normalized extracellular acidification rate (ECAR). *N* = 8. **(G–I)** Relative mRNA **(G)** (Ppargc1a: *p* < 0.001; Nrf1: *p* < 0.001; Tfam: *p* < 0.001) and protein **(H,I)** levels (PGC-1α: *p* = 0.010; NRF1: *p* < 0.001; TFAM: *p* = 0.011) showing activated mitochondrial biogenesis mediated by PGC-1α/NRF1/TFAM pathway after low-dose Propofol treatment. *N* = 3. **(J,K)** Confocal images of PGC-1α (red) staining **(J)** and quantification **(K)** showing an increased intensity of PGC-1α in NSCs in the DG of Nestin-GFP mice 6 h after low-dose Propofol administration at PND 7 in L-Propofol group (*p* = 0.007). *N* = 6. Scale bar, 40 μm. *p < 0.05; **p < 0.01; ***p < 0.001. Error bars, mean ± SD. The Student's *t*-test was used for analysis.

Emerging evidence reveals that mitochondria are the key master regulators in neurogenesis, while the neuronal lineage differentiation of NSCs is highly dependent on mitochondrial OXPHOS (Iwata and Vanderhaeghen, 2021). As the difficulty of metabolism trials performed specifically in hippocampal NSCs *in vivo*, Seahorse XF assays were performed *in vitro* and showed a significant sustained enhancement (2-fold) in oxygen consumption rate (OCR) including basal respiration, maximal respiration, ATP production, spare respiration (Figures 5A–E), and no change in extracellular acidification rate (ECAR; Figure 5F) after low-dose Propofol treatment, verified the activation of OXPHOS in NSCs post-Propofol exposure.

### Low-dose Propofol promoted neurogenesis *via* PGC-1α/NRF1/TFAM pathway

It has been reported that Peroxisome proliferator-activated receptor gamma coactivator 1-alpha (Ppargc1a/PGC-1α) is the central regulator of mitochondrial biogenesis and respiration including OXPHOS and OCR (LeBleu et al., 2014). PGC-1α has been shown to affect neuronal differentiation (Uittenbogaard and Chiaramello, 2014). Therefore, the PGC-1α pathway was investigated after low-dose Propofol treatment. Consequently, increased mRNA level of Ppargc1a was observed to be most pronounced at 6 h post-Propofol treatment both in cultured NSCs (8-fold) (Supplementary Figure 5A) and hippocampus (1.6-fold) (Supplementary Figure 5B). Besides, PGC-1α and its downstream genes including Nuclear Respiratory Factor 1 (NRF1) and mitochondrial transcription factor A (TFAM) (Fanibunda et al., 2019; McMeekin et al., 2021) were all found to be elevated (1.3- to 1.5-fold) by low-dose Propofol at this timepoint *in vitro* (Figures 5G–I). In addition, PGC-1α was also found elevated (1.4-fold) in Nestin^+^ cells of PND7 mice at 6 h-post-Propofol exposure (Figures 5J,K). These data indicated that low-dose Propofol activated PGC-1α-mediated mitochondrial biogenesis, which may exert an important regulatory control on OXPHOS and differentiation in NSCs.

**Figure 6:**
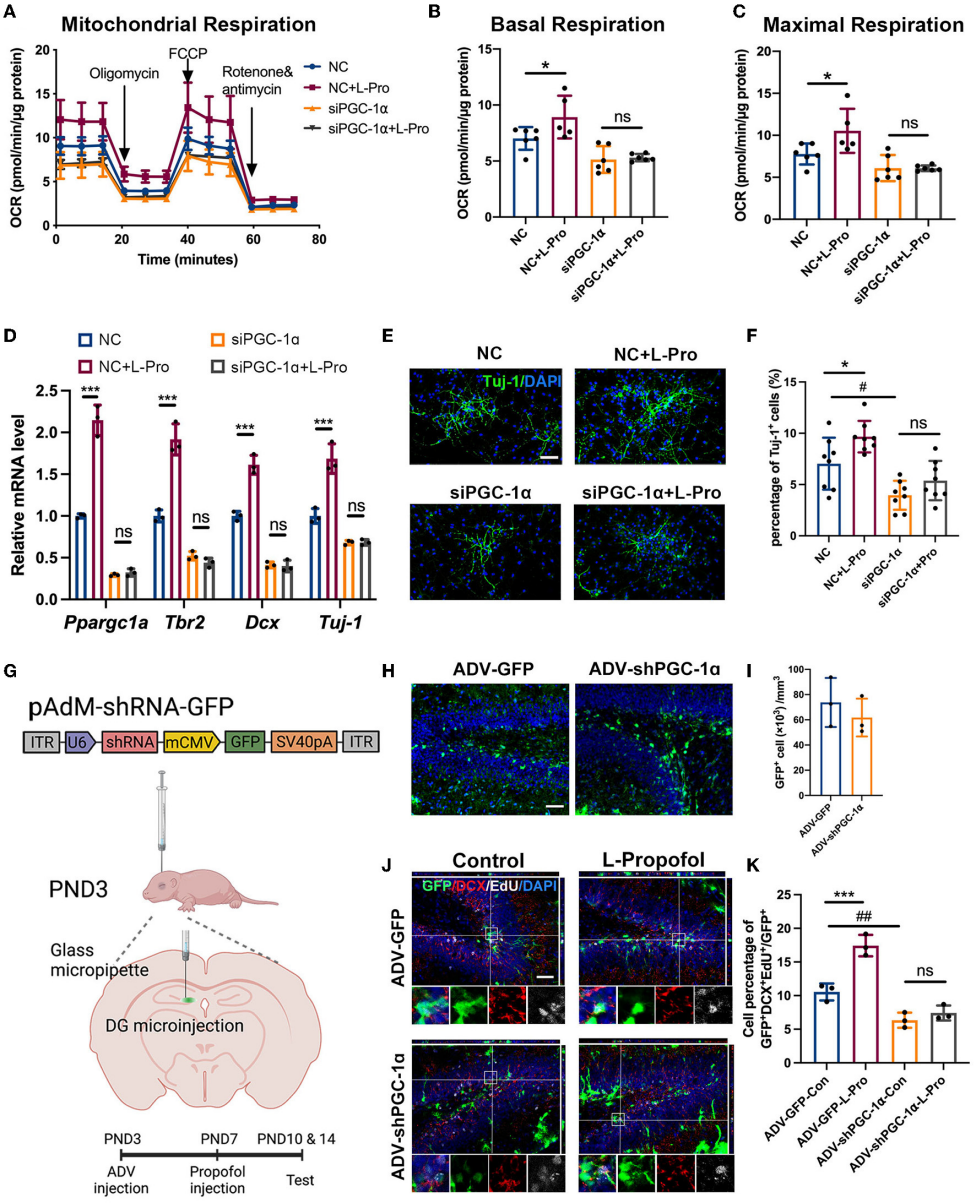
Low-dose Propofol promoted neurogenesis *via* activating PGC-1α-induced mitochondrial OXPHOS. **(A–C)** Administration of siPGC-1α prevented low-dose Propofol-induced mitochondrial OXPHOS promotion in primary NSCs measured by the **(A)** normalized OCR; including **(B)** basal respiration (NC vs. NC+L-Pro: *p* = 0.017, siPGC-1α vs. siPGC-1α+L-Pro: *p* = 0.803) and **(C)** maximal respiration (NC vs. NC+L-Pro: *p* = 0.010, siPGC-1α vs. siPGC-1α+L-Pro, *p* = 0.996), suggesting the dominant role of PGC-1α in Propofol-induced OXPHOS. *N* = 5–6. **(D–F)** siPGC-1α abolished neurogenesis elevation in primary NSCs differentiation determined by **(D)** relative mRNA level (Ppargc1a: NC vs. NC+L-Pro: *p* < 0.001, siPGC-1α vs. siPGC-1α+L-Pro: *p* = 0.979; Tbr2: *p* < 0.001, *p* = 0.776; Dcx: *p* < 0.001, *p* = 0.996), and Tuj-1: *p* < 0.001, *p* > 0.999. *N* = 3) and staining with Tuj-1(green) **(E,F)** (NC vs. NC+L-Pro: *p* = 0.047, siPGC-1α vs. siPGC-1α+L-Pro: *p* = 0.444, NC vs. siPGC-1α: *p* = 0.015. *N* = 8). Scale bar, 50 μm. Error bars, mean ± SD. *(NC vs. NC+L-Pro): *p* < 0.05; *** *p* < 0.001; # (NC vs. siPGC-1α): *p* < 0.05. **(G)** Schematic diagram of stereotactic virus injection. **(H,I)** Confocal images of maximum intensity projection **(H)** and quantification **(I)** of adenovirus infection cell (green) after microinjection in DG in PND10. Scale bar, 50 μm. **(J,K)** Confocal image of EdU (gray) and DCX (red) staining with z-plane projections **(J)** and quantification **(K)** of the percentage of GFP^+^/DCX^+^/EdU^+^ (showing newborn neurons) in GFP^+^ infected cells in the DG of mice at PND 14 after adenovirus injection (ADV-GFP-Con vs. ADV-GFP-L-Pro, *p* < 0.001, ADV-shPGC-1α-Con vs. ADV-shPGC-1α-L-Pro, *p* = 0.334. ADV-GFP-Con vs. ADV-shPGC-1α-Con, *p* = 0.004. *N* = 3). Scale bar, 50 μm. Error bars, mean ± SD. ***(ADV-GFP-Con vs ADV-GFP-L-Pro): *p* < 0.001; *##* (ADV-GFP-Con vs. ADV-shPGC-1α-Con): *p* < 0.01. One-way ANOVA followed by an LSD test was used for analysis.

To substantiate the association between PGC-1α and activated neurogenesis mediated by low-dose Propofol, downregulation of PGC-1α with associated small interfering RNA (siPGC-1α) was conducted in cultured NSCs. Inhibitory effects were evaluated *via* qPCR (70%) *in vitro* (Figure 6D). We found that siPGC-1α significantly prevented the OXPHOS promotion effect of low-dose Propofol (40%) (Figures 6A–C, S6). Besides, siPGC-1α also decreased the expression of neurogenesis markers (Tbr2, Dcx, and Tuj-1) at mRNA level (50–75%) (Figure 6D) and prevented the percentage of Tuj-1^+^ cells (50%) (Figures 6E,F) increased by low-dose Propofol in NSCs differentiation 24 h- and 96 h-induction trials, respectively. We subsequently downregulated PGC-1α using adenovirus (ADV-shPGC-1α) in DG (Figure 6G). The efficiency was evaluated by confirming the infected level (Figures 6H,I) and calculating the downregulated level (57%) of PGC-1α (Supplementary Figure S7) by immunofluorescence staining *in vivo*. As expected, ADV-shPGC-1α injection in DG significantly prevented the elevated newborn neurons (64%) caused by low-dose Propofol (Figures 6J,K). These results provide direct evidence that the PGC-1α pathway participated in low-dose Propofol-activated OXPHOS and neuronal differentiation in NSCs both *in vitro* and *in vivo*.

Taken together, these results indicated the participation of PGC-1α-mediated OXPHOS in neuronal fate commitment preference in NSCs caused by low-dose Propofol.

## Discussion

To explore the effect of Propofol applied in sedation or anesthesia in pediatrics on brain development, we first tested the sub-anesthesia dosage, 4 mg.kg^−1^ (L-Propofol) (Chen et al., 2021) along with the frequently used dosage in mice and 50 mg.kg^−1^ (H-Propofol) in cognitive function of neonatal mice (Jiang et al., 2018). As reported before, under 50 mg.kg^−1^, slightly impaired spatial learning function in the acquisition test was observed (Zhong et al., 2018). Under 4 mg.kg^−1^, mice were sedated but still responding to painful stimuli, and the plasma Propofol concentration was verified as a clinically sedative concentration. Surprisingly, our study demonstrated that mice exhibited a trend of shortened escape latency, and significantly increased platform crossing after low-dose Propofol treatment in the MWM test, suggesting the enhanced spatial cognitive ability. Moreover, we further explored other memory capabilities, including object recognition memory and working memory, as well as the anxiety emotion in mice after Propofol treatment. As no significant difference was found in NOR, Y maze, and OFT, specifically enhanced spatial cognitive abilities after 4 mg.kg^−1^ Propofol treatment was confirmed. Previous studies have reported that low doses of anesthetics presented positive effects on neurodevelopment, such as sevoflurane could promote the learning ability in neonatal rats (Chen et al., 2018). Besides, Propofol at doses of 10–50 mg.kg^−1^ has been reported to protect neurological function in ischemia and hypoxia models (Shi et al., 2014; Yang et al., 2018), but the underlying mechanism has not been fully elucidated. There are still no studies investigating the effects of single low-dose Propofol, which exerts sedative effects, on the developing brain. In this study, 4 mg.kg^−1^ Propofol, which produced the sedative effect in neonatal mice, was used to clarify the effect of low-dose Propofol on children's brain development when undergoing invasive procedures clinically. Our behavioral results demonstrated a new perspective on anesthetics for pediatric brain development: 4 mg.kg^−1^ Propofol promoted cognitive function in neonatal brains.

One of the important factors influencing emotion and spatial cognitive function lies in hippocampal neurogenesis. Decreased neurogenesis in childhood is accompanied by a decline in learning and memory (Doi et al., 2021), while the increase in hippocampal neurons in neonatal mice contributes to the preservation of cognitive function (Martin et al., 2012). In our study, 4 mg.kg^−1^ Propofol increased the overall EdU^+^ newborn cells in the developing hippocampal DG, while lineage tracing also showed significantly increased NSCs expansion and neuronal differentiation. *In vitro*, 10 μM Propofol, closely to plasma concentrations *in vivo* (8 μM) measured in this study (Supplementary Figure 1), was selected and found to significantly increase NSC proliferation and neuronal differentiation. The result was consistent with the conclusion in a previous study that 10 μM Propofol was used to treat human-derived NPC cell lines (Qiao et al., 2017), suggesting that a sedative dose of Propofol promoted NSCs proliferation and neuronal fate decision.

To understand the effect of 4 mg.kg^−1^ Propofol on the hippocampal neurogenesis pool more accurately, an snRNA-seq was adopted to deeply analyze the specific changes in neurogenesis-related cell groups. First, pseudotime analysis suggested a similar pattern of neurogenesis after low-dose Propofol exposure. An increased trend in the proportion of neurons in all differentiation stages was found, especially NPCs (48%), after low-dose Propofol exposure. One possible explanation is that low-dose Propofol not only acted on neurons in a particular stage but might affect neurons at all stages, especially in neuronal differentiation. Another possibility is that Propofol inhibited the apoptosis of newborn neurons, but a previous study showed that apoptosis was not the main factor that Propofol affects the viability of NSCs (Tao et al., 2013), which we confirmed the result in our research (Supplementary Figure 3). Moreover, to observe the safety of Propofol usage in promoting neurogenesis, hippocampal neurogenesis in aged mice after treatment was further evaluated. There was no significant difference in the amount of NSCs in DG after neonatal 4 mg.kg^−1^ Propofol exposure, suggesting the safety of 4 mg.kg^−1^ Propofol exposure in childhood as it would not later cause NSCs pool depletion due to the promotion of neurogenesis. Our study drew consistent conclusions from three aspects: *in vivo* lineage tracing, *in vitro* NSCs experiments, and snRNA-seq, that low-dose Propofol could promote hippocampal neurogenesis in neonatal mice, without causing a significant long-term negative effect.

Mitochondria function plays an essential role in neurogenesis. In healthy PND 3-14 rat brain, PGC-1α, the master regulator of mitochondrial respiration, specifically highly expressed in DG, has been indicated to be important in brain development (Cowell et al., 2007; Finck and Kelly, 2007). By downregulating PGC-1α both *in vitro* and *in vivo*, we found that the promotion of neurogenesis after low-dose Propofol treatment was prevented, implying that PGC-1α-mediated mitochondrial metabolism was of great significance in Propofol-induced neurogenesis. Previous studies have shown that PGC-1α, as a key molecule in mitochondrial biogenesis, is significantly upregulated during NPCs differentiation (Zheng et al., 2016). Besides, it has been reported that overexpression of PGC-1α in NSCs from aged mice affected the proliferation of NSCs *in vitro*, while overexpression of PGC-1α in SVZ of aged mice regulated neural regeneration by increasing DCX^+^ and DA^+^ neurons (Stoll et al., 2015), verifying that PGC-1α could mediate the fate of NSCs. In this study, by downregulating PGC-1α in NSCs, a reduction in the proportion of Tuj-1^+^ cells *in vitro* and a decrease in newborn neurons *in vivo* were observed. Thus, our results suggested that low-dose Propofol could regulate the expression of PGC-1α which could be involved in NSCs neuronal differentiation. However, we did not verify whether overexpression of PGC-1α in NSCs could mimic the pro-neurogenesis phenomenon of low-dose Propofol in this study, which remains to be further explored. Taken together, our results suggested that PGC-1α-mediated mitochondrial metabolism was involved in the effect of low-dose Propofol on hippocampal neurogenesis in neonatal mice.

Propofol is one of the most commonly used drugs for sedation in children. In this study, we explored the effect of 4 mg.kg^−1^ Propofol, as a clinically sedative dosage, on brain development. Our study found that 4 mg.kg^−1^ Propofol promoted hippocampal neurogenesis and spatial cognitive function in neonatal mice. Meanwhile, the crucial role of PGC-1α-mediated mitochondrial metabolism in neurogenesis was further revealed, especially in NSCs' fate decisions. Increasing evidence has shown that mitochondria function contributed to neuronal function protection and neurogenesis regulation. Pharmacological upregulation of PGC-1α expression restores neuronal morphogenesis in mitochondrial dysfunctional cerebral organoids (Inak et al., 2021), while a large number of studies have suggested that PGC-1α is a potential therapeutic target for neurodegenerative diseases (Panes et al., 2022). Thus, our work provides new clues for the usage of low-dose Propofol in pediatrics examinations as a potential benefit for neurodevelopment in the vulnerable period.

## Data availability statement

The datasets in the current study are available from the corresponding author on reasonable request. Raw data and processed data of snRNA-seq are available at the Gene Expression Omnibus (GEO) database with accession number “GEO: GSE186216”. Raw data and processed data of bulk RNA-seq are available at the GEO database with accession number “GEO: GSE200968”.

## Ethics statement

The animal study was reviewed and approved by Ethical Committee of Sun Yat-sen University.

## Author contributions

XF and MW: study supervision. KC, DL, and XY: study design/planning. KC, DL, XY, RZ, LL, YW, XX, CW, and MHJ: study conduct. All authors contributed to the article and approved the submitted version.

## Funding

This study was supported by the National Natural Science Foundation of China [Grant Numbers: 81770290 (MHJ), 81870829 (XF), 82071224 (XF), and 31972894 (MHJ)], the Science and Technology Program of Guangzhou, China (201804010492, XF), and the Major Project of Basic and Applied Basic Research Foundation of Guangdong Province (2019B1515120054, XF).

## Conflict of interest

The authors declare that the research was conducted in the absence of any commercial or financial relationships that could be construed as a potential conflict of interest.

## Publisher's note

All claims expressed in this article are solely those of the authors and do not necessarily represent those of their affiliated organizations, or those of the publisher, the editors and the reviewers. Any product that may be evaluated in this article, or claim that may be made by its manufacturer, is not guaranteed or endorsed by the publisher.
