# Supplementary material for: Enhanced hippocampal neurogenesis mediated by PGC-1α-activated OXPHOS after neonatal low-dose Propofol exposure
Source: Front Aging Neurosci. 2022 Jul 27;14:925728. doi: 10.3389/fnagi.2022.925728 (PMC9363786; doi:10.3389/fnagi.2022.925728)
Supplement: Supplementary file 1 [file Data_Sheet_1.docx]

**Supplemental Information**

**Supplemental Data**


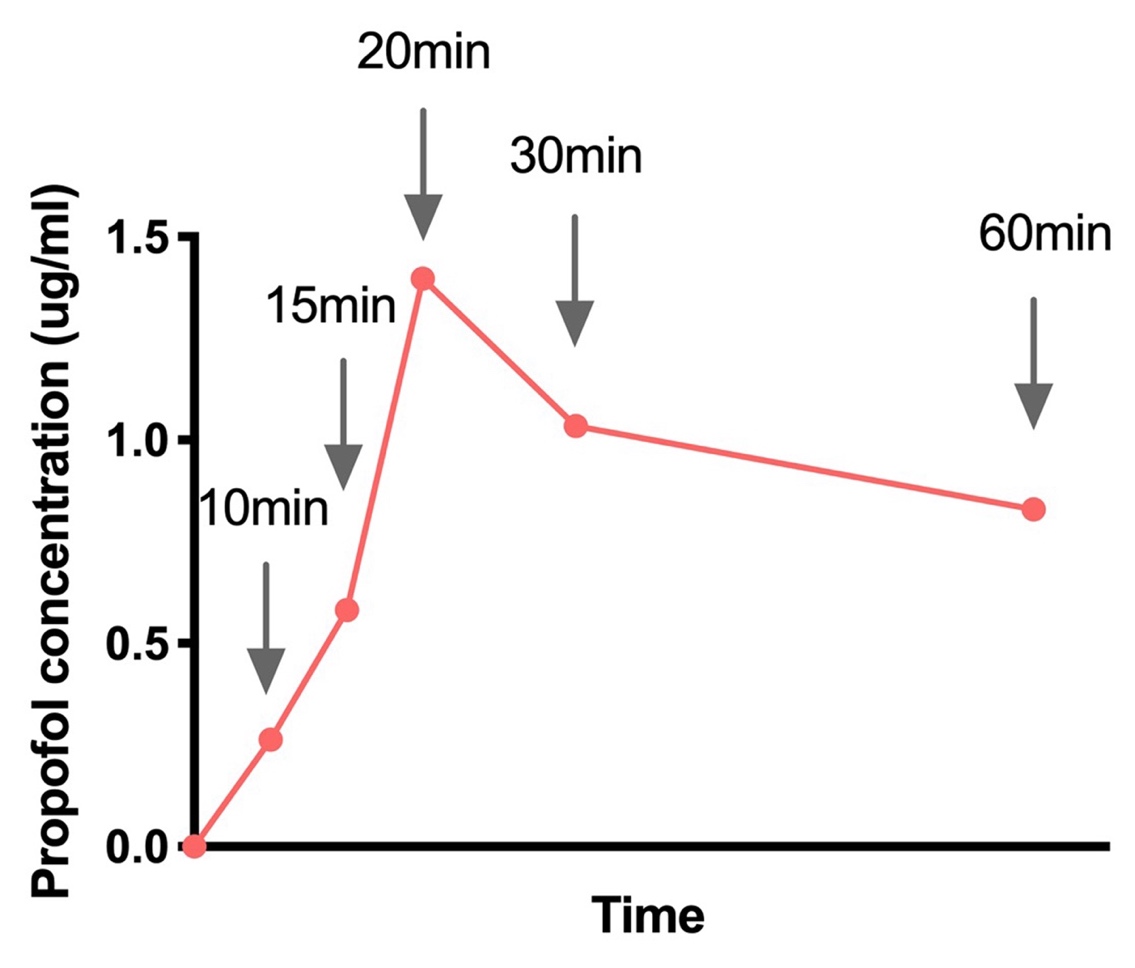


**Fig S1. The plasma concentration of Propofol after i.p. injection of 4 mg.kg^-1^ Propofol.**

The plasma concentration of Propofol evaluated by HPLC in the first hour after i.p. injection of 4 mg.kg^-1^ Propofol.

**
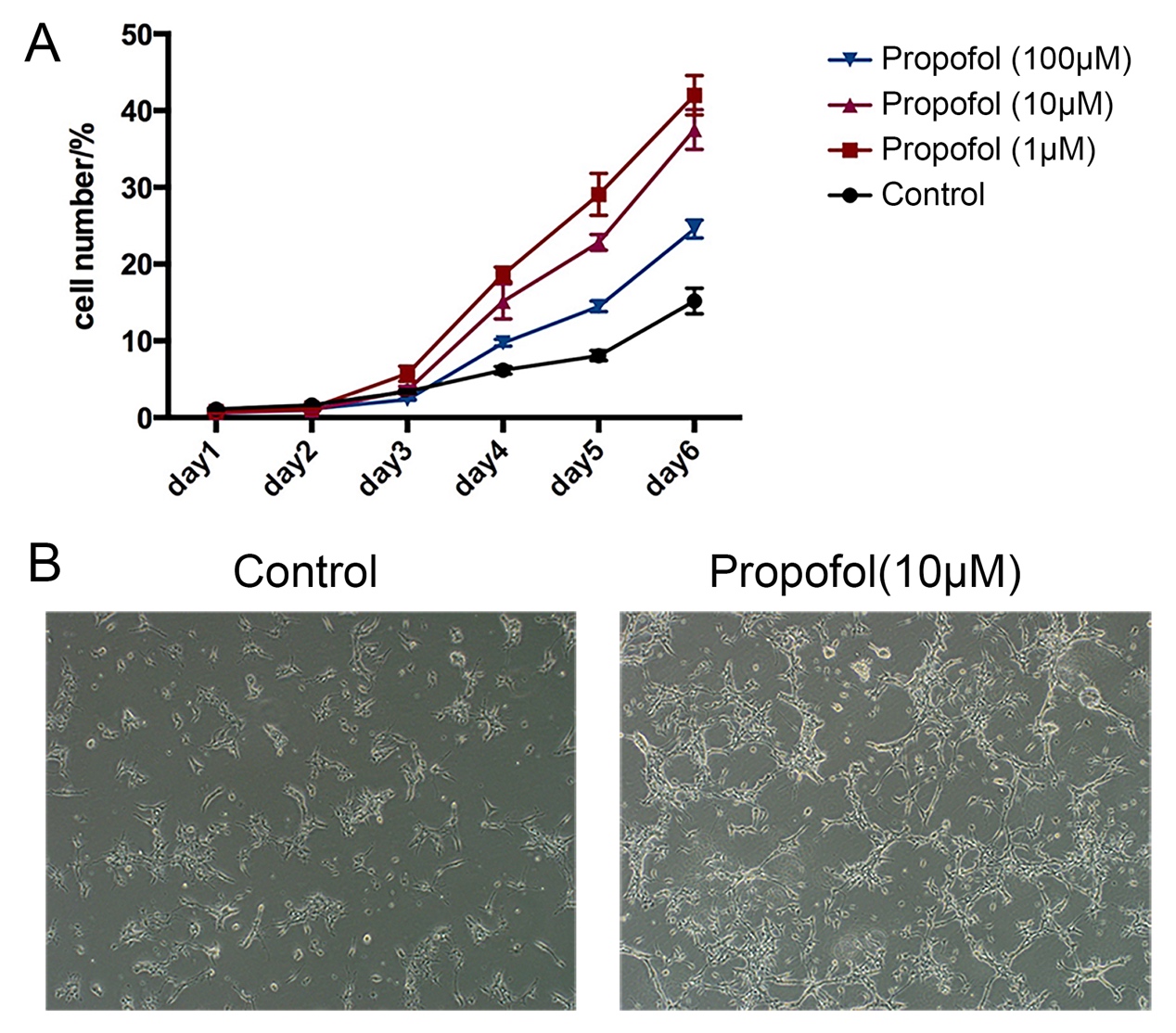
**

**Fig S2. Effect of different concentrations of Propofol on NSCs proliferation.**

**A** Growth curves of NSCs as assessed by direct counting after treatment with different concentrations of Propofol. N=3. Error bars, mean ± SD. **B** Representative images in bright field showing NSCs growth after 24 hours-treatment in Propofol at 10μM.

**
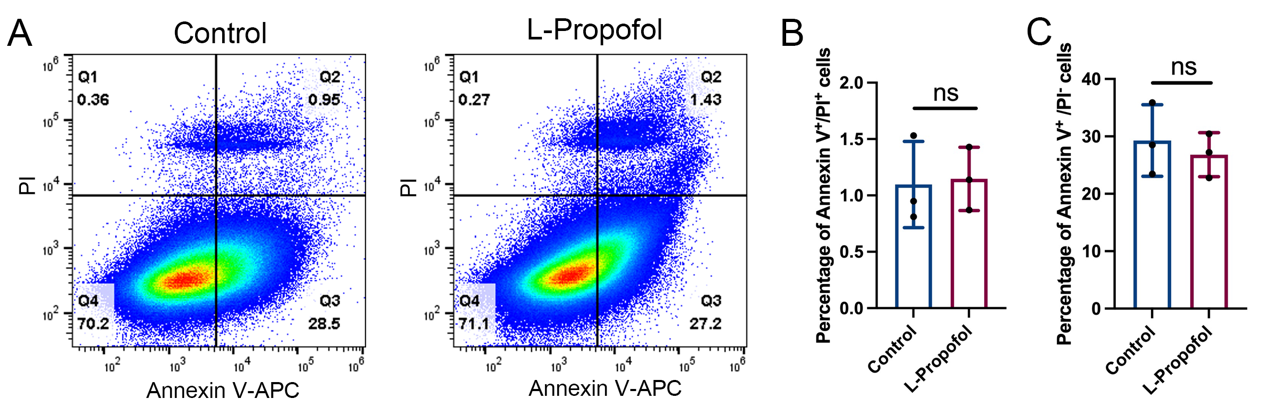
**

**Fig S3. Apoptosis assessment after low-dose Propofol treatment in NSCs *in vitro*.**

**A** Representative experiment in Annexin V/PI Flow cytometry analysis post-treatment of low-dose Propofol. Q1 quadrant represents percentage of necrotic cells, Q2 quadrant represents percentage of late apoptotic cells. Q3 quadrant represent percentage of early apoptotic cells. Q4 quadrant represent percentage of viable cells. **B, C** Quantification of the percentage of late apoptotic cells (B) and early apoptotic cells (C). N=3, Error bars, mean ± SD. ns=no significant. Student’s t-test was used for analysis.

**
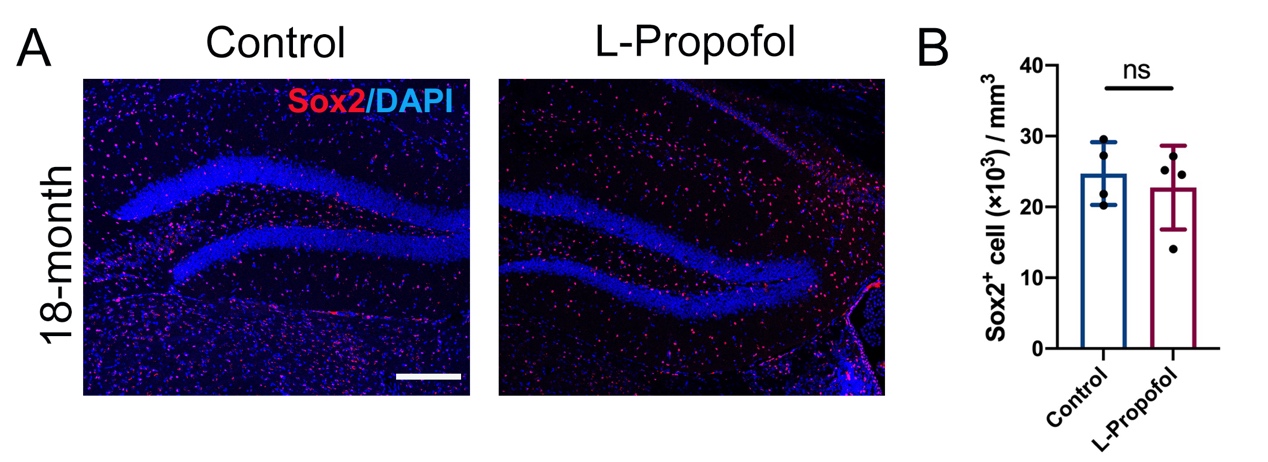
**

**Fig S4. Similar neurogenesis level in aged mice after neonatal exposed to low-dose Propofol.** Confocal image of Sox2 (Red) staining (**A**) and quantification of Sox2^+^ cells (**B**) in DG of mice at 18-month. N=4. Scale bar, 200μm. Error bars, mean ± SD, ns=no significant. Student’s t-test was used for analysis.

**
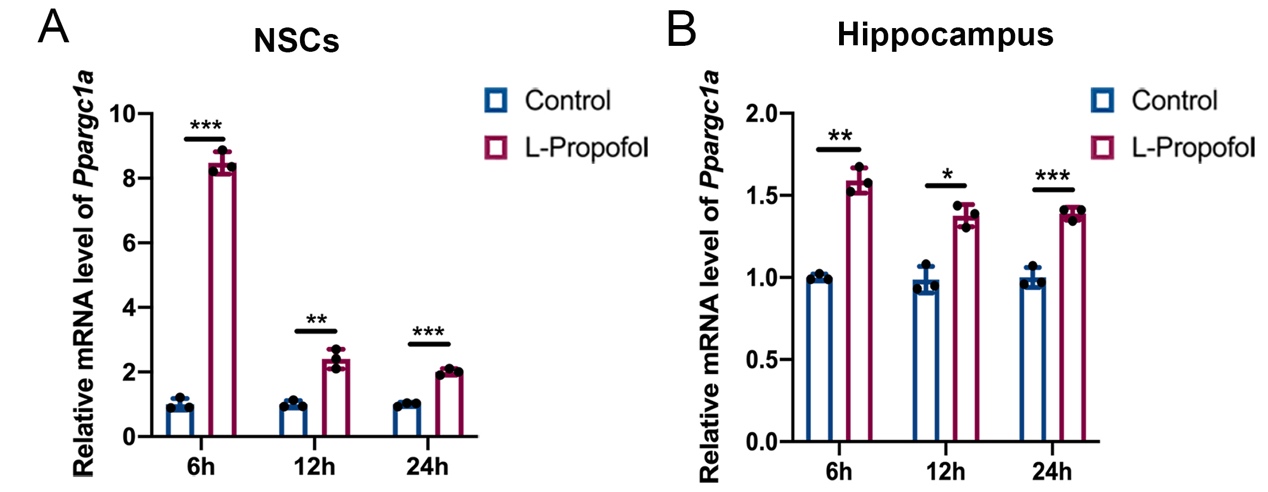
**

**Fig S5. *Ppargc1a* levels assessed at different timepoints after Propofol administration in primary cultured NSCs (A) and the hippocampus of PND7 mice (B).** Relative mRNA level of *Ppargc1a* was significantly increased most 6h after Propofol exposed compared with 12h and 24h both in NSCs and hippocampus. N=3. Error bars, mean ± SD. *: *p*<0.05, **: *p*<0.01, ***: *p*<0.001. Student’s t-test was used for analysis.


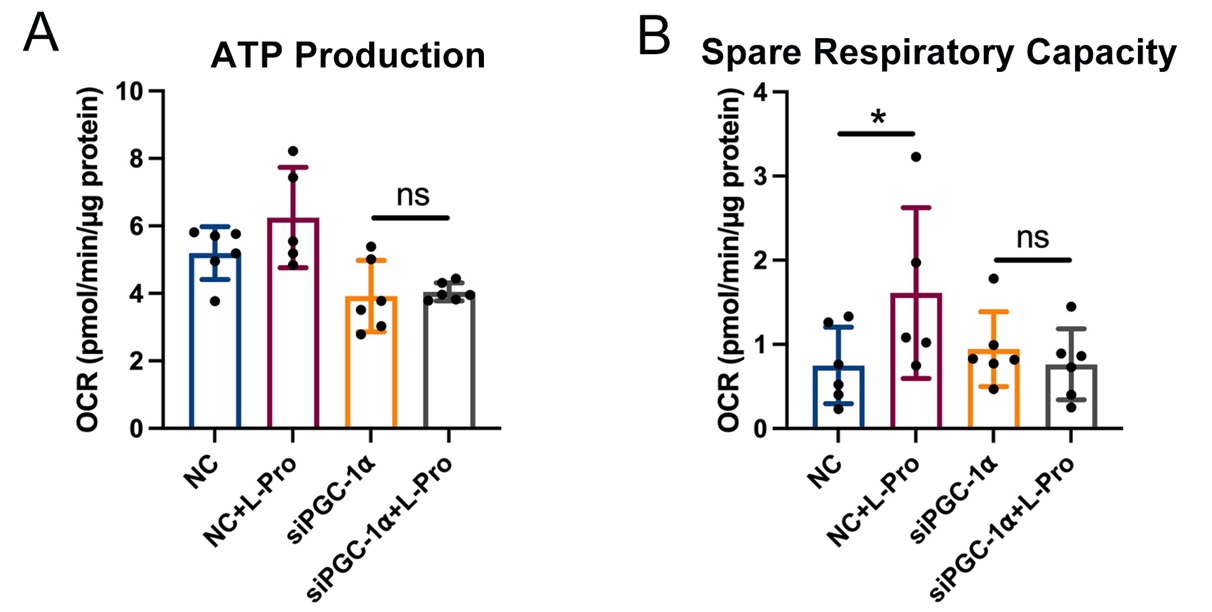


**Fig S6. siPGC-1α prevented the OXPHOS promotion effect of low-dose Propofol in normalized OCR of ATP production (A) and Spare respiratory capacity (B).** N=5-6, Error bars, mean ± SD. *: *p*<0.05. ns=no significant. One-way ANOVA followed by LSD test were used for analysis.


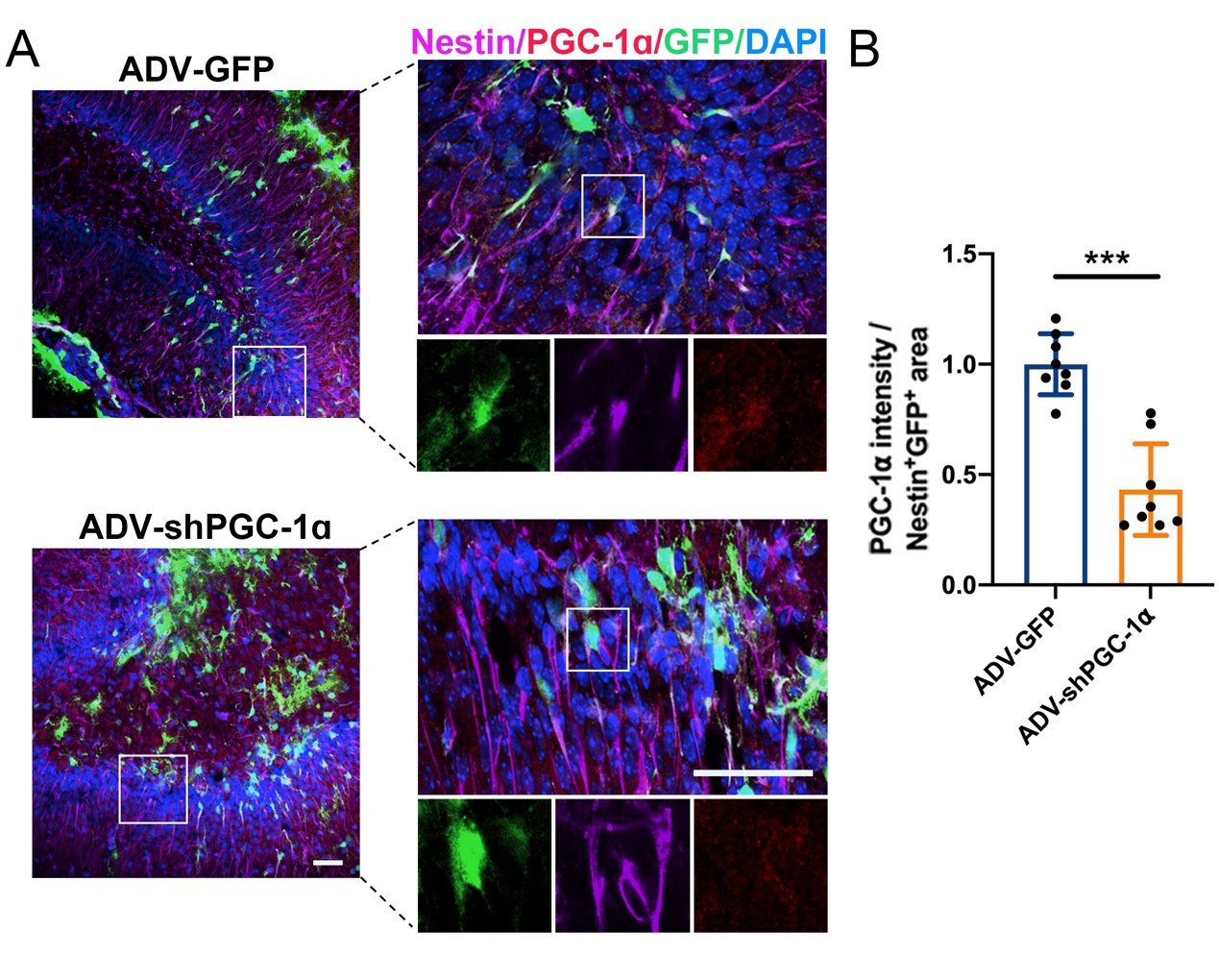


**Fig S7. Evaluation of PGC-1ɑ knock down effect in NSCs in hippocampus**

Confocal images (**A**) of low magnification (left), high magnification (right) and quantification (**B**) of the level of PGC-1⍺ down-regulation (*p*<0.001) by staining with PGC-1⍺ (red), Nestin (purple) in DG in PND10 after ADV (green) injection. N=8, Scale bar, 50μm. Error bars, mean ± SD, ***: *p*<0.001. Student’s t-test was used for analysis.

**Supplemental Methods**

**Behavioral test**

Behavioral tests were performed 4 weeks after drug injection.

Morris water maze (MWM) was carried out in a pool of 122 cm in diameter and 51 cm in height with water filled with non-toxic tempura paint. Mice were released into the pool with platform submerged below the water. All the mice underwent 3-day acquisition experiments, and each mouse performed 4 trials each day at the same time period. The probe trials were performed 24 hours after the last training day with platform removed. Mice were allowed to swim for 90 s and mice's behaviors were recorded by a video camera (Panasonic, Osaka, Japan) connected to the computer with Water Maze Tracking System (MT-200; Chengdu TME Technology Co, Ltd; Chengdu, China). The spatial learning and memory ability were estimated by escape latency and swimming distance in acquisition experiments, as well as the number of times crossing the platform in the probe trials.

Anxiety behavior tested by open field test (OFT) was performed in an open field device (40 cm × 40 cm × 40 cm). After habituated to the test room, the mouse was placed into the middle part of empty chamber and stayed for 10 minutes. The behaviors of experimental mice were recorded and analyzed by a video camera and the Clever Sys., Inc. software (Clever Sis, Virginia, America). The anxiety degree was estimated by the anxiety index, which calculated based on the proportion of time spent in the central region/ time spent in the total region.

For novel object recognition test (NOR), the experiment was carried out in the same devices as above described in OFT. During the object recognition task, mice were first placed in the chamber and exposed to two identical objects for 10 minutes (sample phase). Then mice returned to its cage for a 2-hour delay. Then mice were placed back in the chamber for another 10 minutes exposed to a familiar object and a novel object which placed at the same location as the previous object (choice phase). The time that mice spent in exploring objects were recorded. The cognition function was estimated by discrimination ratio, which was calculated based on the proportion of time spent exploring the novel object/ total time spent exploring both objects in the choice phase.

Y-maze test was performed in a Y maze consisted of three arms (5 cm × 35 cm × 15 cm) with an angle of 120˚ between each arm. Each mouse was released through the start arm into the maze with all arms open to navigate freely all over the arms for 5 min. Arm entry was defined as entry of the whole body into an arm. The sequence of arm entry was recorded. The spatial working memory was estimated by alternation index, which was calculated based on the proportion of number of consecutive entries into all three arms/ number of arm entries.

**Immunoﬂuorescence Staining and Quantification**

For tissue immunofluorescent staining, after being anesthetized with 50mg.kg^-1^ 2% pentobarbital, the mice were perfused with 0.9% saline followed by 4% paraformaldehyde (PFA; AR1068; BOSTER; California, USA). The whole brain was dissected out and fixed in 4% PFA for 24h before being transferred to 30% sucrose at 4°C for another 48 h. The brains were frozen at -20 °C with OCT (Optimal Cutting Temperature; 4583; SAKURA; Tokyo, Japan) and cryosectioned coronally at 30 μm with a microtome (SM1950; Leica; Wetzlar, Germany). Brain sections and cells were permeabilized in 0.25% Triton X-100 (T8787; Sigma-Aldrich; Missouri, United States) for 15 min and blocked with normal goat serum in TBS for 40 min, and then stained with primary antibodies Nestin, NeuN, Sox2 overnight at 4℃ or DCX 12h at room temperature, following by incubation with secondary antibodies including anti-mouse IgG and anti-rabbit IgG protect from light at room temperature for 1 h after washing three times for 5 min in TBS/T. EdU staining was performed following the instructions of the EdU imaging kit (C10637, C10634; Invitrogen; California, United States). Images were obtained using Confocal laser scanning microscope (LSM780, 880, ZEISS, Oberkochen, Germany; Dragonfly, Andor, United Kingdom) with z-stack. The volume of section was calculated by measuring the DAPI area in the image and multiplying the measured area with the number of z-axis slices and the distance between slices (1-µm-interval). Staining positive cells within the section were counted manually. Staining intensity were obtained from single confocal z-planes by Image J software.

For cell immunofluorescence, cells were fixed in 4% PFA. Staining process was same with brain section immunofluorescence. Images were obtained using Confocal laser scanning microscope, microscope (DMi8; Leica; Wetzlar, Germany) with single plane. Quantification was determined by marker positive cells in total DAPI^+^ cells.

**Apoptosis analysis**

After drug treatment for 24h, the cells were collected and resuspended with 100μl binding buffer. Cell density in the cell suspension was adjusted to 5×10^3^ cells.μl^-1^. Subsequently, 5μl Annexin V-FITC was added to the cell suspension followed by gentle vortexing and incubation for 10 min at room temperature in the dark. Thereafter, the cell suspension was incubated with 5μl Propidium iodide. All procedures followed the instructions of the commercial kit (556547; BD Bioscience). Cells were analyzed using flow cytometry (Gallios; Beckman Coulter) for Annexin V-FITC and Propidiumiodide binding immediately. Data were analyzed with FlowJo version 10.1 software (BD, Ashland, USA).

**RNA isolation, Reverse Transcription and Quantitative Real-Time PCR (qRT-PCR)**

Cells or hippocampus were sorted into TRIzol reagent (15596018; Invitrogen) and total RNA was extracted. A total of 1 μg of RNA was reverse transcribed using the RevertAid First Strand cDNA Synthesis Kit (K1622; Thermo Scientific; California, United States). The cDNA thus obtained was subjected to real-time PCR with the SYBR Green reagent (32057220; Roche; Basel, Switzerland). Quantitative real-time PCR was performed as described elsewhere. Mouse primers used are listed in Table S1. The relative mRNA abundance was calculated using the ∆Ct or ∆∆Ct methods, and gene expression levels were normalized to those of Gapdh.

**Western blot analysis**

Total proteins were extracted by 1× RIPA lysis buffer (20-188; Millipore; California, United States) with protease inhibitor cocktail (4693116001; Roche, Basel, Switzerland), and the protein concentration was measured using a BCA protein assay kit (23227; Thermo Scientific). The proteins were separated using 7.5% or 10% sulfate-poly-acrylamide gel electrophoresis and then transferred to a 0.45-μm polyvinylidene fluoride (PVDF) membrane (Millipore); the membrane was then blocked with Tris-buffered saline TBS/T containing 5% Bovine Serum Albumin (BSA) and analyzed for the target proteins. The antibodies used are listed in Table S3.

**HPLC Analysis**

Plasma was obtained from PND7 mice after i.p. injection of Propofol. Each timepoint contained the samples from 6 neonatal mice. Samples were analyzed by high-performance liquid chromatography (HPLC, Agilent 1200 RRLC; Agilent Technologies; California, United States) coupled with mass spectrometry (Agilent 6410; Agilent Technologies). HPLC separation system was fitted with a C18 column (Proshell120 EC-C18; 3.0 mm × 30 mm, 2.7 µm; Agilent Technologies) from Agilent Technologies. The gradient elution was formed by 0.01% formic acid in methanol as mobile phase A, and 0.1% formic acid in water as mobile phase B. The flow rate was kept at 0.3 mL/min. The column temperature was kept at 25°C and the injection volume was 5 µL. Mass analysis was performed in a SIM mode as the following: the fragmentor voltage was 120V, the drying gas temperature was 325℃, the drying gas flow was 10 L/min, and the Nebulizer was 25psi.

**Seahorse metabolism analysis**

NSCs were seeded 5 × 10^4^ cells at each well of XFe96 cell culture plates with PLL coated and incubated at 37 °C in a 5% CO2 incubator for 24 h until they were fully attached to the plate. Cells were than treated with 10μΜ Propofol for 24 h and then send to Seahorse XF96 Analyzer (Agilent Technologies) for tests. To determine mitochondrial respiratory chain activity, cells were washed and incubated in 180 μL Seahorse XF base media (102353-100, Agilent Technologies) with 1mM pyruvate (S8638, Sigma), 10 mM glucose (G8769, Sigma), 2 mM L-glutamine (G8540, Sigma) in a non-CO2 incubator for 1 h at 37 °C. After measurement of basal oxygen consumption rates, cells were sequentially treated with oligomycin A (1 μM), FCCP (1 μM), rotenone (0.5 μM) and antimycin A (0.5 μM). For glycolysis stress test, cells were washed and incubated in 180 μL XF base media with 1 mM L-glutamine in a non-CO2 incubator for 1 h at 37 °C. After determination of basal ECAR, cells were sequentially treated with glucose (10 mM), oligomycin (1 μM), 2-DG (50 mM). The total protein of each well was determined by Bradford assay and used to normalize the oxygen consumption rate or ECAR.

**Supplemental Tables**

**Table S1. Primers for qRT-PCR analysis**

| ID | Primer name | Accession number | Primer sequence（5’ to 3’） |
| --- | --- | --- | --- |
| 1 | *Ppargc1a* forward | NM_008904 | GAATCAAGCCACTACAGACACCG |
| 2 | *Ppargc1a* reverse | NM_008904 | CATCCCTCTTGAGCCTTTCGTG |
| 3 | *Nrf1* forward | NM 010938 | GGCAACAGTAGCCACATTGGCT |
| 4 | *Nrf1* reverse | NM 010938 | GTCTGGATGGTCATTTCACCGC |
| 5 | *Tfam* forward | NM_009360 | GAGGCAAAGGATGATTCGGCTC |
| 6 | *Tfam* reverse | NM_009360 | CGAATCCTATCATCTTTAGCAAGC |
| 7 | *Gapdh* forward | [NM_008084](https://www.ncbi.nlm.nih.gov/nuccore/NM_008084) | CATCACTGCCACCCAGAAGACTG |
| 8 | *Gapdh* reverse | [NM_008084](https://www.ncbi.nlm.nih.gov/nuccore/NM_008084) | ATGCCAGTGAGCTTCCCGTTCAG |

**Table S2. Antibodies for Immunohistochemistry and Immunoblotting**

| **Primary/Secondary antibody** | **Company** | **Catalog number** | **Detail** |
| --- | --- | --- | --- |
| Nestin | Abcam,  Cambridge, UK | ab11306 | Immunohistochemistry 1:100 |
| NeuN | Abcam,  Cambridge, UK | ab104224 | Immunohistochemistry 1:200 |
| DCX | Abcam,  Cambridge, UK | ab18723 | Immunohistochemistry 1:100 |
| Tuj-1 | Abcam,  Cambridge, UK | ab14545 | Immunoﬂuorescence 1:200 |
| GFAP | Abcam,  Cambridge, UK | ab7260 | Immunoﬂuorescence 1:200 |
| Sox2 | Invitrogen,  California, US | 14-9811-82 | Immunoﬂuorescence 1:500 |
| PGC-1α | Abcam,  Cambridge, UK | ab54481 | Immunohistochemistry 1:500 |
| PGC-1α | Santa cruz  California, US | sc-518025 | Immunoblotting 1:200 |
| NRF1 | CST,  Massachusetts, US | 46743 | Immunoblotting 1:1000 |
| TFAM | Abcam,  Cambridge, UK | Ab47517 | Immunoblotting 1:1000 |
| GAPDH | CST  Massachusetts, US | 5174 | Immunoblotting 1:1000 |
| Anti-mouse IgG (Alexa Fluor® 488 Conjugate) | CST,  Massachusetts, US | 4408 | Immunoﬂuorescence 1:500 |
| Anti-rabbit IgG (Alexa Fluor® 488 Conjugate) | CST,  Massachusetts, US | 4412 | Immunoﬂuorescence 1:500 |
| Anti-mouse IgG (Alexa Fluor® 594 Conjugate) | CST,  Massachusetts, US | 8890 | Immunoﬂuorescence 1:500 |
| Anti-rabbit IgG (Alexa Fluor® 555 Conjugate) | CST,  Massachusetts, US | 4413 | Immunoﬂuorescence 1:500 |
| Anti-mouse IgG (Alexa Fluor® 647 Conjugate) | Invitrogen,  Carlsbad, US | A32728 | Immunoﬂuorescence 1:1000 |
| Horseradish peroxidase-conjugated anti-mouse | CST,  Massachusetts, US | 7076 | Immunoblotting 1:3000 |
| Horseradish peroxidase-conjugated anti-rabbit | CST,  Massachusetts, US | 7074 | Immunoblotting 1:3000 |
